# Weakly-Emergent Strain-Dependent Properties of High Field Superconductors

**DOI:** 10.1038/s41598-019-50266-1

**Published:** 2019-09-30

**Authors:** Paul Branch, Yeekin Tsui, Kozo Osamura, Damian P. Hampshire

**Affiliations:** 1 University of Durham, Superconductivity Group, Department of Physics, Durham, DH1 3LE UK; 2 Research Institute of Applied Sciences, Kyoto, 6068202 Japan

**Keywords:** Materials science, Physics

## Abstract

All superconductors in high field magnets operating above 12 T are brittle and subjected to large strains because of the differential thermal contraction between component parts on cool-down and the large Lorentz forces produced in operation. The continuous scientific requirement for higher magnetic fields in superconducting energy-efficient magnets means we must understand and control the high sensitivity of critical current density *J*_c_ to strain *ε*. Here we present very detailed *J*_c_(*B*, *θ*, *T*, *ε*) measurements on a high temperature superconductor (HTS), a (Rare−Earth)Ba_2_Cu_3_O_7−*δ*_ (REBCO) coated conductor, and a low temperature superconductor (LTS), a Nb_3_Sn wire, that include the very widely observed inverted parabolic strain dependence for *J*_c_(*ε*). The canonical explanation for the parabolic strain dependence of *J*_c_ in LTS wires attributes it to an angular average of an underlying intrinsic parabolic single crystal response. It assigns optimal superconducting critical parameters to the unstrained state which implies that *J*_c_(*ε*) should reach its peak value at a single strain (*ε* = *ε*_peak_), independent of field *B*, and temperature *T*. However, consistent with a new analysis, the high field measurements reported here provide a clear signature for weakly-emergent behaviour, namely *ε*_peak_ is markedly *B*, (field angle *θ* for the HTS) and *T* dependent in both materials. The strain dependence of *J*_c_ in these materials is termed weakly-emergent because it is not qualitatively similar to the strain dependence of *J*_c_ of any of their underlying component parts, but is amenable to calculation. We conclude that *J*_c_(*ε*) is an emergent property in both REBCO and Nb_3_Sn conductors and that for the LTS Nb_3_Sn conductor, the emergent behaviour is not consistent with the long-standing canonical explanation for *J*_c_(*ε*).

## Introduction

The critical current density *J*_c_ is the maximum current density that can be carried by a superconductor before significant dissipation results from flux flow. It is the most important parameter in high field magnet design for systems such as MRI^1^, particle accelerators^2^ and fusion energy reactors^3^. In high field superconductors, *J*_c_ is usually parameterised in terms of a volume flux pinning force *F*_p_ and is a function of magnetic field *B*, temperature *T*, applied uniaxial strain *ε*_app_ and for an anisotropic conductor, the angle *θ* between *B* and say the normal to the tape surface.

There are various theories of flux pinning that describe *J*_c_. Theory^4,5^ and experiment^6,7^ often lead to the same generalised scaling law of the form

1 $${F}_{{\rm{p}}}={J}_{{\rm{c}}}B=A\frac{{B}_{{\rm{c}}2}^{n}}{{(2\pi {\varphi }_{0})}^{\frac{1}{2}}{\mu }_{0}{\kappa }_{1}^{m}}{b}^{p}{(1-b)}^{q},$$

where *B*_c2_ is the upper critical field, *κ*_1_ is the Ginzburg-Landau parameter, *b* = *B*/*B*_c2_ is the reduced field, *μ*_0_ is the vacuum permeability, *ϕ*_0_ is the magnetic flux quantum, *A* is a material dependent constant, and *n*, *m*, *p* and *q* are constants dependent on the specific pinning mechanism operating. The flux pinning scaling law is widely observed in many different types of superconducting materials including low temperature superconductors (LTS) and high temperature superconductors (HTS). This is because its form is derived using Ginzburg-Landau theory, which is founded on Landau’s very general theory of second-order phase transitions, and can equally well describe superconductors with different microscopic fundamental mechanisms causing the superconductivity^8^. The standard literature uses Eq. (1) to describe field (angle), temperature, and strain dependencies^7,9,10^. The temperature and strain dependencies of *J*_c_ are a result of the dependencies of *B*_c2_, *T*_c_ and *κ*_1_, where *T*_c_ is the critical temperature. The field dependence is determined by the constants *p* and *q* where a change of 1/2 indicates a change in pinning mechanism^4^. When *n* = 5/2, *m* = 2, *p* = 1/2 and *q* = 2, *A* ≈ 1/250 in the LTS Nb_3_Al^7^ and *A* ≈ 1/100 in Nb_3_Sn^10^. For HTS, such as the (RE)Ba_2_Cu_3_O_7−*δ*_ (REBCO, RE = Rare Earth) conductor presented here, *B*_c2_ in Eq. (1) is associated with the irreversibility field *B*_irr_ (a field below *B*_c2_ where *J*_c_ falls to zero but the material is still in the superconducting state).

Technological high field superconductors are designed to have as high a *J*_c_ as possible and hence strong pinning. This leads to them being complex and inhomogeneous, so we rarely find the integral and half-integral values of *n*, *p* and *q* given in simple flux pinning theory^4^. In this paper we do not add to the huge literature that considers the range of pinning mechanisms that operate in HTS and LTS materials^9–13^. We simply use the scaling law and the derived parameters as a convenient way to characterise the field, angle and temperature dependencies accurately, but as we shall see, not the strain *ε*.

Commercial conductors, of the type measured here are complex composites, where typically, *J*_c_ has an inverted parabolic applied strain dependence with a peak value when *ε* = *ε*_peak_^14–16^. The differential thermal contraction between the different parts of the conductor during cool-down means that although the intrinsic strain on the superconducting component itself (*ε*_int_) can be very different from the applied strain (*ε*_app_), in many REBCO tapes and Nb_3_Sn wires, *ε*_peak_ is found to occur when the intrinsic strain *ε*_int_ is approximately zero^17,18^. Hence both compressive and tensile strain are generally considered to degrade the superconducting properties of high field conductors.

The magnetic field (angle) and temperature dependencies of *J*_c_ have been extensively reported for most high field technological superconductors using commercial or well-established magnetometers and transport measuring equipment^19^. One can consider the scaling law as a starting point, or a framework, for describing the functional dependencies of *J*_c_. Here we call the scaling law ‘primary’ along with the associated parameters when it is used to describe the *B* and *T* dependencies of the whole material. Although *J*_c_(*B*, *T*) is controlled by many mechanisms, Eq. (1) can be considered primary because measurements of the material provide averages of the critical underlying distribution of qualitatively similar components within the material, be that the bulk material or domains themselves in single crystalline type HTS materials, or the grain boundaries in polycrystalline LTS materials^14^. We use primary in the same sense that it is used in say primary legislation which describes broad principles and is usually underpinned by many detailed laws that are all qualitatively similar^20^. In contrast, uniaxial strain measurements that include both compressive and tensile strain measurements are much less common and are currently made using bespoke equipment. Here we show that the strain dependence of *J*_c_(*ε*) is best understood and described as weakly-emergent. It emerges from a competition between component parts. We define emergent properties as those properties of the whole material that are not qualitatively similar to equivalent properties of the underlying component parts^21^. We add ‘weakly’ to contrast this type of emergence from emergence that cannot be predicted even with a detailed knowledge of the component parts. Our definition of weakly-emergent properties includes for example the well-known motion of a flock of birds^22^ or a colony of ants^23^ where there is no centralised decision making, but simple local rules obeyed by an individual can in principle be measured and, with sufficient computation, shown to lead to the complex overall behaviour^24^. The strain dependence of *J*_c_(*ε*) is usefully described as emergent because the overall behaviour is qualitatively very different to that of its components and weakly-so because, as shown in this paper, we can calculate *J*_c_(*ε*). This description also helps highlight for the reader, that there has been the decades-long incorrect treatment of the magnetic field and temperature dependencies on an equal footing to the strain dependence.

Soon after the discovery of HTS, Dimos showed that *J*_c_ was low in polycrystalline materials because of high-angle grain boundaries^25^. This led to many studies of the strain dependence of the grain boundary *J*_c_(*ε*)^26–29^ and subsequently the technological development of HTS conductors of the type investigated here. Such conductors typically consist of a tape ~100 *μ*m thick, 4 mm wide and a superconducting layer that only accounts for a few percent of the cross sectional area. The superconducting layer in our REBCO tape is a quasi-single crystal such that the *c*-axis is approximately normal to the tape surface and grain misorientation angles are minimised^30^. It is twinned along the {110} planes so there are some domains where the *a*-axis is aligned along the direction of the tape (domain A) and the remaining domains have the *b*-axis (domain B) aligned. The strain dependence of the critical parameters of single crystal REBCO is well known^31^, showing anisotropy with respect to applied strain. The critical temperature *T*_c_ shows a linear increase with tension along the *a*-axis, a linear decrease of the same order along the *b*-axis and is insensitive to strain applied along the *c*-axis. Hence the well-defined twinned microstructure in REBCO conductors that includes domains with opposite strain dependencies led Van der Laan *et al*. in 2011 to point out a qualitative correlation between anisotropic single crystal behaviour and *J*_c_(*ε*) in conductors^32^. Subsequently Osamura *et. al*. developed a bimodal chain model that was quantitatively consistent with zero field *J*_c_(*ε*) data^33^. Here we report extensive *J*_c_(*B*, *θ*, *T*, *ε*) measurements on a REBCO HTS tape and develop the analysis sufficiently to describe the more important in-field behaviour. In contrast to the standard assumption that *ε*_peak_ is a constant, we find experimentally that *ε*_peak_ is a marked function of *B*, *θ* and *T* and conclude from the theoretical analysis that it occurs because of a competition between different components of the HTS with opposite monotonic strain dependencies. Hence we identify a clear signature for emergent rather than primary behaviour and provide a quantitative analysis that gives the relative populations and properties of the component parts that compete.

Here we also report extensive *J*_c_(*B*, *T*, *ε*) measurements on an A15 LTS state-of-the-art bronze-route Nb_3_Sn wire manufactured by Bruker for use in the ITER fusion energy reactor^3^. These data also display the commonly observed inverted parabolic strain behaviour of *J*_c_(*ε*)^7,10^. Historically, the degradation of *J*_c_ with strain in LTS, was discovered at a time when A15 materials held the record value for *T*_c_ (i.e. pre-1986). The canonical explanation for the strain dependence of *T*_c_ in A15 compounds follows ab-initio calculations that are consistent with a coincidence between the Fermi energy and a peak in the density of states produced by the narrow *d*-band electrons in the Nb-chains^34^. In principle, this explains the relatively high values of *T*_c_, and the optimum values of *J*_c_ occuring in the unstrained or zero intrinsic strain state (i.e. *ε*_int_ = 0)^35^. The strain dependency of *T*_c_ is attributed to variations in both phononic and electronic properties. In this canonical description the parameter *ε*_peak_ specifies the optimum strain state, or equivalently the optimum atomic spacings in the material, for peak superconducting critical parameters such as *T*_c_, and therefore should not depend on *B* and *T*. Given the very good scaling of *F*_p_, it has also been assumed since then that all the material responds to an applied strain in a similar manner and hence measurements of *J*_c_ provided averaged properties^6,7,15^. However even now, although Nb_3_Sn is to be used in the multi-billion dollar ITER fusion tokamak^36^ and the LHC high-luminosity upgrade^37^, uniaxial strain dependent single crystal data (for Nb_3_Sn^38^ or any A15 material^39^) remain very limited. We have found that even in the limited data available, there is no experimental evidence for the optimum superconducting critical properties in single crystals occurring in the unstrained state. This undermines the generally accepted interpretation of *ε*_peak_ that includes equating the strain dependent properties of polycrystalline Nb_3_Sn wires, such as *T*_c_ and *B*_c2_, to an angular average of single crystal properties^14^. We propose that although *ε*_peak_ is the optimum strain for the overall properties of the material, one has to abandon the standard interpretation that *ε*_peak_ is associated with the optimum properties for the component parts of the material. High *J*_c_ wires of the type presented here are designed for high field operation. This makes them prone to instability in low fields and in practice has prevented any reports of experimental data describing the strain dependence of high *J*_c_ Nb_3_Sn in zero-field. Here we present high field measurements and find, strikingly, that as with REBCO, *ε*_peak_ is a marked function of *B* and *T*. Hence we conclude that *J*_c_(*ε*) in both REBCO and Nb_3_Sn is emergent.

## Methods

**Figure 1:**
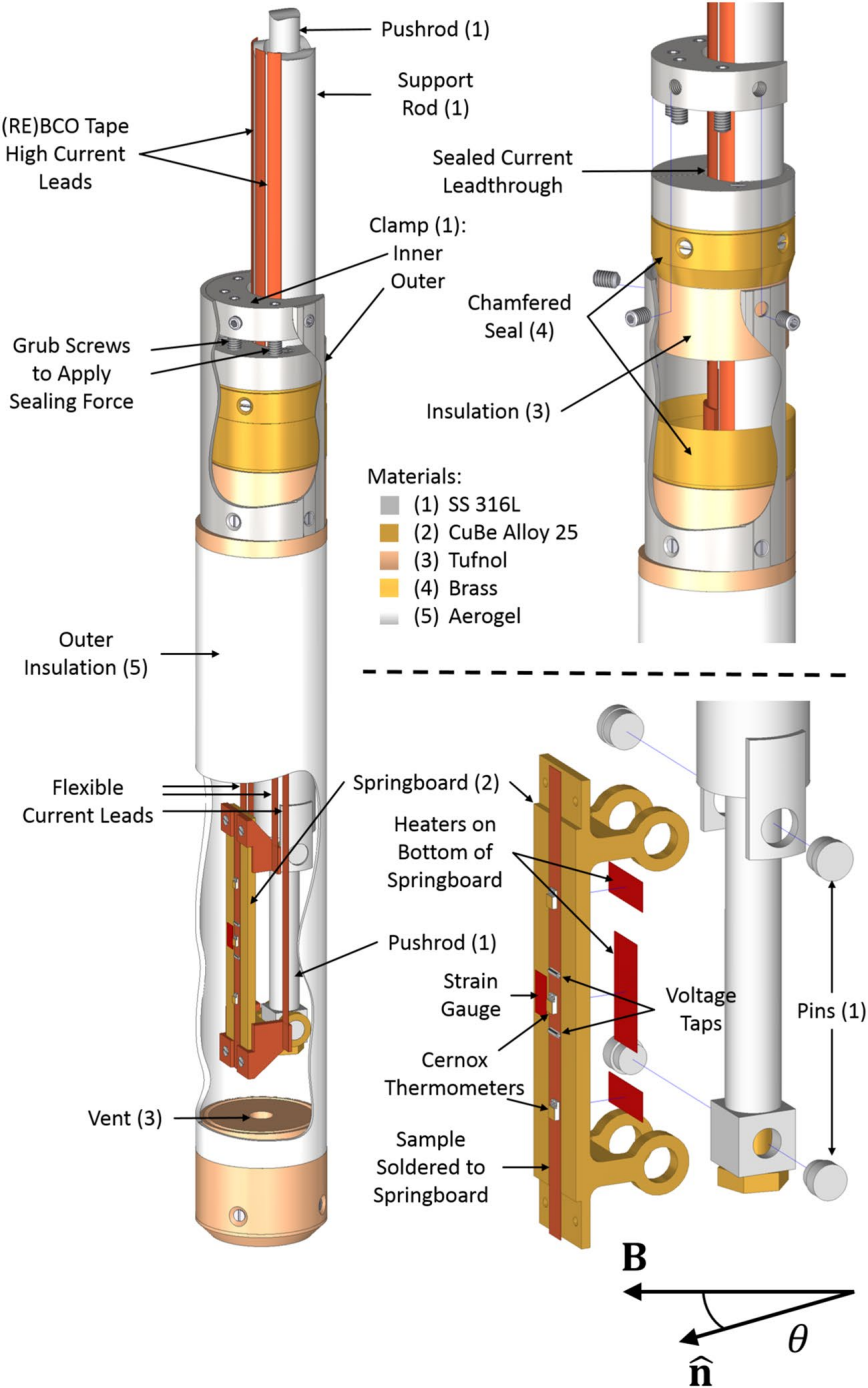
Schematic of the bottom of the measurement probe used for REBCO. The numbers in brackets specify the material used to fabricate the component.

Transport *J*_c_ and *B*_c2_ measurements were performed on a HTS REBCO coated conductor manufactured by SuperPower^40^ (Ref: SCS4050) using the four-probe method with a custom-built probe in our in-house 15 T liquid helium cooled, 40 mm wet-bore, superconducting, split-pair horizontal magnet^41^. The sample was soldered to the top of a springboard made of CuBe as shown in Fig. 1. Compressive and tensile strain can be applied to the sample by pulling apart or pushing together the legs of the springboard. Force was applied to the legs of the springboard using a pushrod attached to a screw jack with a high gearing ratio. The strain was monitored continuously using a strain gauge attached to the springboard alongside the sample next to the voltage taps. The voltage tap separation was 13 mm, located about the centre of the springboard. Temperature control was achieved through use of an inverted temperature cup^42^. The cup is sealed at the top and has a vent at the bottom as shown in Fig. 1. Initially it fills with liquid helium. Three heaters attached to the underside of the springboard drive the liquid helium out through the vent leaving a gaseous environment. The temperature of the sample was controlled by a temperature controller using the three heaters in conjunction with three field calibrated Cernox^TM^ resistance thermometers attached to the top, the middle and the bottom of the sample. The field calibration for the thermometry was taken from literature^43^ and confirmed in liquid helium at 4.2 K.

**Figure 2:**
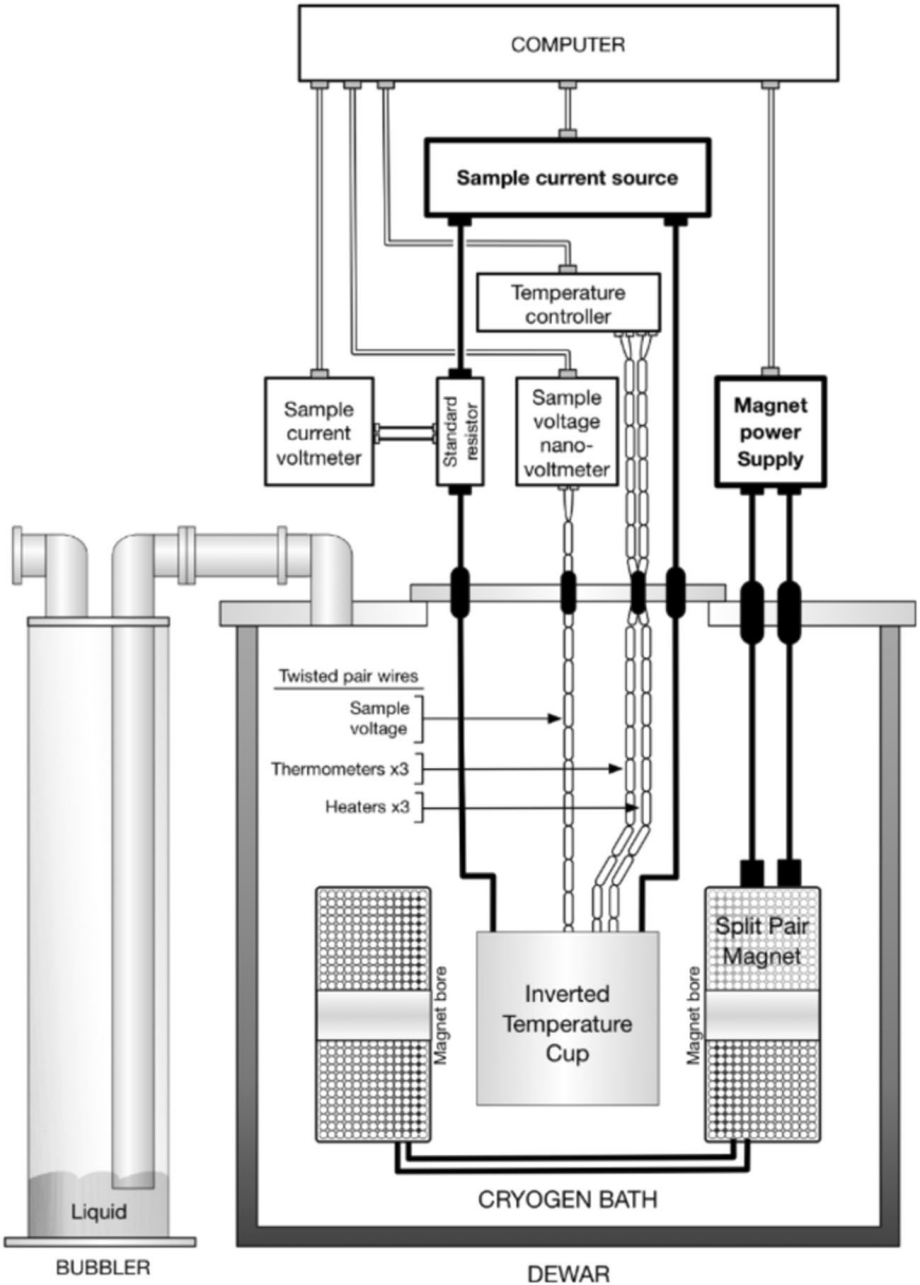
Schematic showing the hardware used for making critical current and resistivity measurements as a function of field, field-angle, temperature and strain^19^.

*J*_c_ measurements were performed holding the field, temperature and strain constant, and ramping the current at a rate such that each measurement took ~60 s. The voltage, current and temperature were measured continuously. A nanovolt amplifier with a gain of 50,000 was used to amplify the voltage signal and the current was determined by measuring the voltage drop across a calibrated low resistance shunt connected in series with the power supply and sample. The experimental setup is shown in Fig. 2. The current through the superconductor *I*_SC_ is slightly lower than that supplied by the power supply *I*_total_ due to current shunting through the sample holder and stabilising materials in the conductor. This was accounted for by subtracting the shunt current from the measured current using the equation

2 $${I}_{{\rm{SC}}}={I}_{{\rm{total}}}-\frac{V}{{R}_{{\rm{shunt}}}},$$

where *V* is the measured voltage across the sample, and *R*_shunt_ is the resistance of the sample holder and stabilising materials which was determined as a function of field and strain from the *B*_c2_ traces. The typical magnitude of the shunt current was 80 mA at 100 *μ*V m^−1^. The critical current was converted to a critical current density using the cross-sectional area of the superconductor, taken to be 4 × 10^−3^ mm^2^. *J*_c_ was determined at a critical *E*-field criterion of 100 *μ*Vm^−1^, and the index of transition *N* by fitting the relation *E* ∝ *J*^*N*^ between 10 and 100 *μ*V m^−1^.

*B*_c2_ measurements were performed holding the field and strain constant. A small current of 100 mA was applied and the temperature increased to above the transition at a rate of 1 Kmin^−1^. The voltage and temperature were measured continuously and *B*_c2_ was determined at the onset of the superconducting transition (i.e. close to 100 % of the normal state resistance of the stabilising matrix of the composite).

The sample was aligned with respect to the magnetic field using a Hall probe attached to the sample such that *θ* = 0° when the magnetic field was normal to the surface of the tape. Measurements were taken first at *θ* = 0°. The strain was taken to *ε*_app_ = −1 % and held constant as *J*_c_ and *B*_c2_ were obtained as a function of field and temperature. At temperatures of 4.2, 20, 40 and 60 K measurements of *J*_c_ were taken from 2 to 14 T in intervals of 2 T or until *I*_total_ > 250 A (the maximum current the probe can sustain). At temperatures of 68 and 76 K measurements were taken at 1 T intervals up to 14 T or until *B* > *B*_c2_. *B*_c2_ measurements were taken at fields of 0 to 14 T in intervals of 2 T. The strain was then increased in intervals of 0.25 % to +0.5 % and held constant at each strain where another field and temperature dependent dataset was obtained. To ensure the sample was undamaged by the strain cycle, eventually the applied strain was relaxed to zero and measurements of *J*_c_ at 2 T and 60 K, and *B*_c2_ at 2 T were taken and were found to agree with the results taken at the start of the experiment.

Dense *J*_c_ measurements were then taken as a function of angle to complement the data taken at fixed angle. The peak in *J*_c_, when the field is aligned with the *ab*-plane, was found at *θ* = 87.5° showing there was a −2.5° difference between the *ab*-plane and the tape surface. The dense angular measurements were used to select four angles at which to perform detailed strain dependent measurements *θ* = 47.5°, 77.5°, 82.5° and 87.5° which cover a large range in *J*_c_. The strain was taken to *ε*_app_ = −1 % and held as *J*_c_ measurements were obtained as a function of angle, at temperatures of 20, 40 and 60 K and fields from 2 to 14 T in intervals of 2 T or until *I*_total_ > 250 A. The strain was then increased in intervals of 0.25 % to +0.5 % and held at each strain where another field, temperature and angle dependent dataset was obtained. Again the strain was relaxed and measurements of *J*_c_ and *B*_c2_ taken and were found to agree with the previous results showing the sample remained undamaged.

The very high values of *B*_c2_ in REBCO mean it was not possible to measure it directly at low temperatures. The lack of data at high reduced field in the low temperature region also meant it was not possible to determine *B*_c2_ using the universal flux pinning scaling curve (as is the case with the Nb_3_Sn sample). To obtain *B*_c2_ at low temperatures we first established the universal flux pinning scaling in the high temperature region at *θ* = 0° (*T* = 60, 68 and 76 K) using the directly measured values of *B*_c2_. The parameters *p* and *q* were then fixed at the values obtained from the high temperature data, and the *J*_c_ data in the low temperature region at *θ* = 0° (*T* = 4.2, 20 and 40 K) and all temperatures at *θ* ≠ 0° were fitted to the universal flux pinning curve allowing *B*_c2_ to be a free parameter.

Transport *J*_c_ data were also taken on a LTS bronze-route Nb_3_Sn wire using the four-probe method with a custom-built probe in an in-house 17 T liquid helium cooled, 40 mm wet-bore, superconducting, vertical solenoid magnet^44^. The field was applied orthogonal to the axis of the wire. Strain was applied to the sample using a Walters spring. Measurements of *J*_c_ were taken from *ε*_app_ = −1.16 % to *ε*_app_ = +0.58 %, at temperatures of *T* = 4.2, 8, 10, 12, 14 K and various fields chosen such that typically eight in-field measurements were taken at each combination of temperature and strain. Direct transport measurements of *B*_c2_ were not obtained for this sample. *B*_c2_ was determined from the field at which the pinning force density fell to zero in the universal pinning curve.

## Results and Analysis

**Figure 3:**
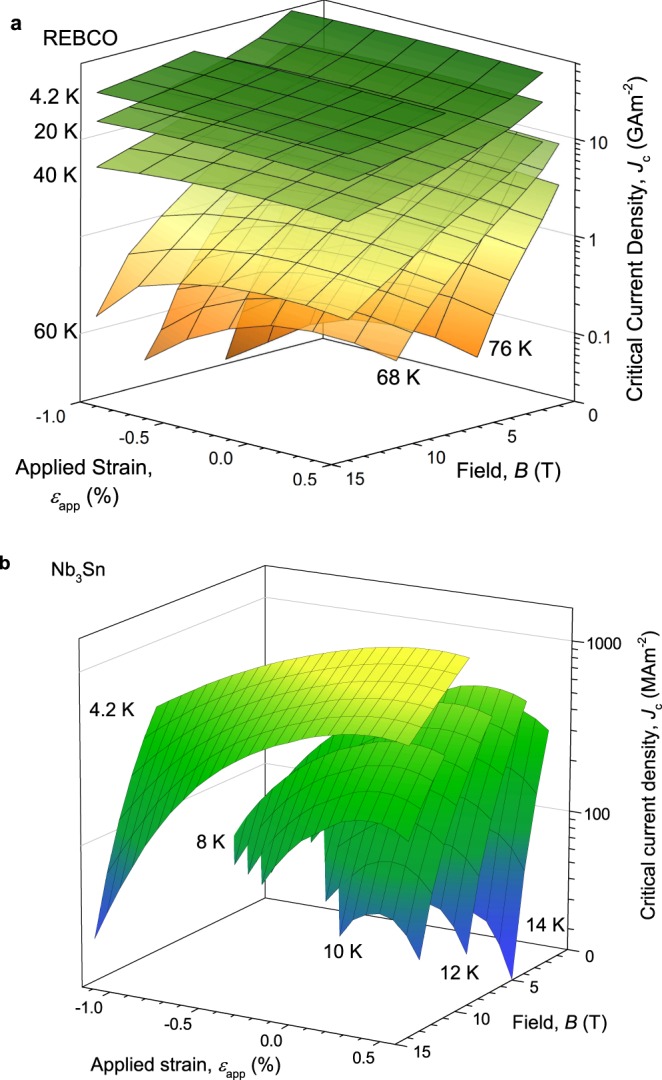
Critical current density data for (**a**) REBCO coated conductor at *θ* = 0° and (**b**) bronze route Nb_3_Sn. Both materials show inverted parabolic behaviour as a function of strain.

**Figure 4:**
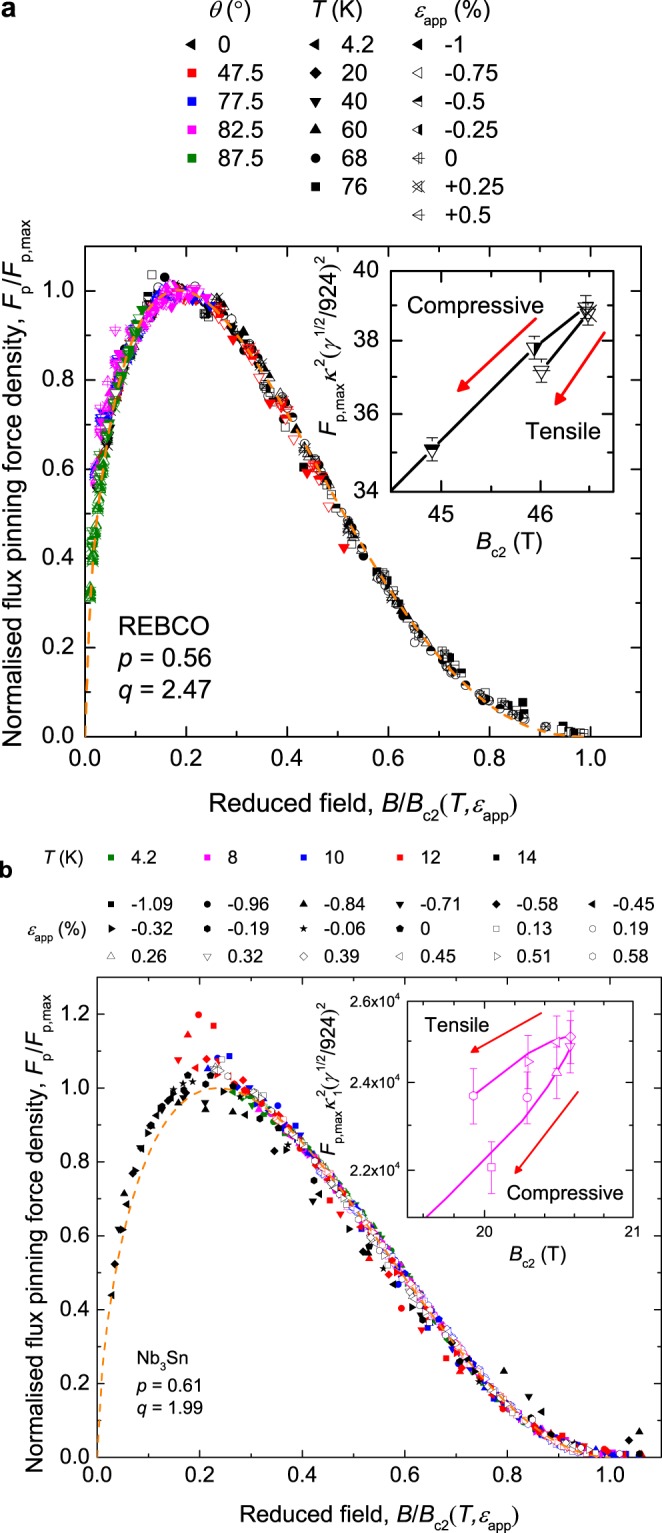
Universal temperature and strain independent flux pinning scaling curves for (**a**) REBCO coated conductor and (**b**) bronze route Nb_3_Sn wire. (insets) ${F}_{{\rm{p}},{\rm{\max }}}{\kappa }_{1}^{2}$ against *B*_c2_ on a log-log scale at (**a**) 40 K for REBCO and (**b**) 8 K for Nb_3_Sn, showing double-valued behaviour.

Figure 3 shows our extensive field *B*, temperature *T* and strain *ε* dependent set of transport *J*_c_ and *B*_c2_ measurements on REBCO where the field was applied orthogonal to the flat surface of the tape and to the Nb_3_Sn wire axis. Figure 4 shows the universal scaling of the normalised pinning force versus the normalised magnetic field for both samples. Additional *J*_c_ data for the HTS conductor are included in Fig. 4 for different angles *θ*. The insets show that some of the scatter on the universal curves is associated with ${F}_{{\rm{p}},{\rm{\max }}}{\kappa }_{1}^{2}$ being double-valued such that its value in tension is not equal to that in compression for the same *B*_c2_, where we have taken *m* = 2 and *κ*_1_ = 924*B*_c2_/*γ*^1/2^*T*_c_(1 − *t*^2^) where *t* = *T*/*T*_c_ is the reduced temperature^10^.

The bimodal chain model developed for zero field data considers the tape as a chain of domains A and B with relative domain fractions *f* and (1 − *f* ) respectively^33^. Under strain, the superconducting properties of one domain increase while those in the other domain decrease. At the highest tensile or compressive strains, *J*_c_ of the tape is dominated by just one of the domains, namely that with the lowest *J*_c_. By considering the tape as a 1D twinned single crystal, the model attributes the inverted parabolic nature of the strain dependence of *J*_c_ to the competition between the two domains with opposite strain dependencies. This interpretation is in contrast to the standard explanation that attributes the inverted parabolic response of *J*_c_(*ε*) in LTS conductors to the intrinsic averaged behaviour of the underlying material.

**Figure 5:**
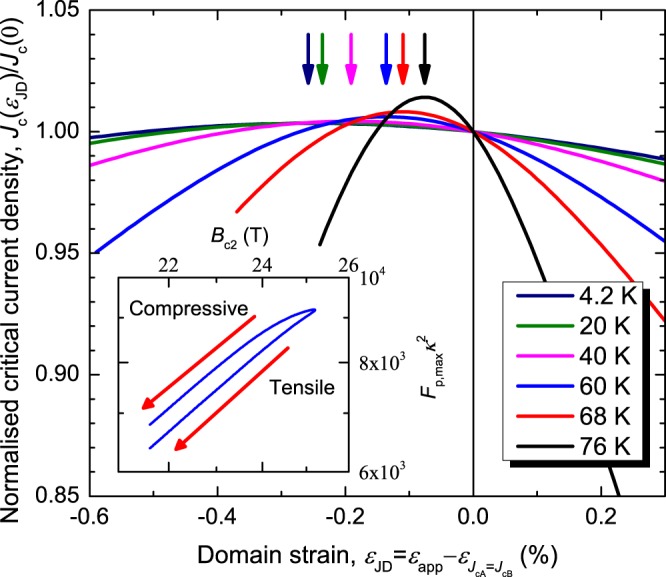
Numerical results of the bimodal model for REBCO at *B* = 5 T with *f* = 0.3 showing the normalised critical current density against the domain strain as a function of temperature. *ε*_peak_, the strain at which *J*_c_ reaches a maximum, is indicated by the arrows. For *f* ≠ 0.5, *ε*_peak_ is a function of field and temperature. (inset) Numerical results of the bimodal model for REBCO showing the double-valued behaviour of ${F}_{{\rm{p}},{\rm{\max }}}{\kappa }_{1}^{2}$ as a function of *B*_c2_ on a log-log scale at 60 K. Both the field and temperature dependence of *ε*_peak_ and the double-valued behaviour of ${F}_{{\rm{p}},{\rm{\max }}}{\kappa }_{1}^{2}$ require bimodal behaviour with competing domains and do not appear in models that consider *J*_c_(*ε*) as primary rather than emergent, or when *f* = 0.5.

In the analysis here, we distinguish those features in the bimodal model that are not present in models that attribute *J*_c_(*ε*) to a single component. This identifies the emergent properties of *J*_c_(*ε*). The electric field *E* generated by a bimodal system is given by

3 $$E={E}_{{\rm{c}}}f{(\frac{J}{{J}_{{\rm{cA}}}})}^{{N}_{{\rm{A}}}}+{E}_{{\rm{c}}}(1-f){(\frac{J}{{J}_{{\rm{cB}}}})}^{{N}_{{\rm{B}}}},$$

where *E*_c_ is the electric field criterion for *J*_c_, *N*_*i*_ is the index of transition in domain *i* and is defined through the empirical relation ${N}_{i}=1+r{J}_{{\rm{c}}i}^{s}$ where *r* and *s* are material dependent constants^45^ and *J*_c*i*_, the critical current density in domain *i*, is of the form of Eq. (1) where *i* = *A* or *B*. In general, the strain tensor is not the same in both domains so we introduce a new strain called the domain strain ${\varepsilon }_{{\rm{JD}}}={\varepsilon }_{{\rm{app}}}-{\varepsilon }_{{J}_{{\rm{cA}}}={J}_{{\rm{cB}}}}$ where ${\varepsilon }_{{J}_{{\rm{cA}}}={J}_{{\rm{cB}}}}$ is the applied strain at which *J*_c*i*_ in both domains is equal and is independent of field and temperature. The conditions for the tape to carry its critical current density are *J* = *J*_c_ and *E* = *E*_c_ which in general means Eq. (3) is transcendental in *J*_c_. Figure 5 shows numerical solutions for *J*_c_, including the temperature (and magnetic field not shown) dependence of *ε*_peak_ and the double-valued behaviour of ${F}_{{\rm{p}},{\rm{\max }}}{\kappa }_{1}^{2}$ when *f* = 0.3. The strain dependencies of *J*_c*i*_ are introduced by assuming the strain dependence of *T*_c_ in each domain is linear with opposing strain dependencies, consistent with single crystal data^31^. In general, when *f* < 0.5, as in Fig. 5, ${\varepsilon }_{{\rm{peak}}} < {\varepsilon }_{{J}_{{\rm{cA}}}={J}_{{\rm{cB}}}}$, taking lower values at lower temperatures, and ${F}_{{\rm{p}},{\rm{\max }}}{\kappa }_{1}^{2}$ is lower in tension than compression for the same value of *B*_c2_. When *f* = 0.5 the behaviour of *J*_c_ is indistinguishable from homogeneous models and ${\varepsilon }_{{\rm{peak}}}={\varepsilon }_{{J}_{{\rm{cA}}}={J}_{{\rm{cB}}}}$. When *f* > 0.5 then ${\varepsilon }_{{\rm{peak}}} > {\varepsilon }_{{J}_{{\rm{cA}}}={J}_{{\rm{cB}}}}$, taking higher values at lower temperatures, and ${F}_{{\rm{p}},{\rm{\max }}}{\kappa }_{1}^{2}$ is higher in tension than compression for the same value of *B*_c2_. We conclude that if *f* ≠ 0.5, *ε*_peak_ is field, temperature and *f* dependent which cannot be accounted for by models where measurements are attributed to an averaged or homogeneous underlying material. Also, ${F}_{{\rm{p}},{\rm{\max }}}{\kappa }_{1}^{2}$ is a weakly double-valued function of *B*_c2_ although this can also be explained in a homogeneous model if ${B}_{{\rm{c}}2}(0,\varepsilon )$ is not a single valued function of *T*_c_(*ε*).

We now calculate approximate values of *f* and ${\varepsilon }_{{J}_{{\rm{cA}}}={J}_{{\rm{cB}}}}$ for the HTS and LTS samples by deriving an analytic form for *ε*_peak_. For small changes in strain, we can take *J*_c*i*_ to have a linear strain response which is equal and opposite in each domain

4 $${J}_{{\rm{c}}i}({\varepsilon }_{{\rm{JD}}})=\{\begin{array}{cc}{J}_{{\rm{c}}i}(B,T,0)(1+g{\varepsilon }_{{\rm{JD}}}) & i={\rm{A}},\\ {J}_{{\rm{c}}i}(B,T,0)(1-g{\varepsilon }_{{\rm{JD}}}) & i={\rm{B}},\end{array}$$

where *g* is a function of temperature and field calculated by taking a first order Taylor expansion of Eq. (1) in strain about *ε*_JD_ = 0 %. Equation 4 has the form that follows from the assumption that the field and temperature dependence of *J*_c*i*_ in both domains is the same. *ε*_peak_ is then calculated as the turning point of a second order Taylor expansion of Eq. (3) about *ε*_JD_ = 0 % to give

5 $${\varepsilon }_{{\rm{peak}}}=\frac{2f-1}{f(1-f)}\frac{F({N}_{0},s)}{g(B,T)}+{\varepsilon }_{{J}_{{\rm{cA}}}={J}_{{\rm{cB}}}},$$

where *F* = *N*_0_/(*N*_0_(*N*_0_ + 1) − 2*s*(*N*_0_ − 1)) and *N*_0_ is the index of transition at *ε*_JD_ = 0%. There is typically ~10 % difference between the analytic Eq. (5) and the numerical results in Fig. 5. The functional form of *g* is dependent on the parameterisation of *B*_c2_ which is different for the HTS REBCO and LTS Nb_3_Sn samples. For the REBCO sample, *B*_c2_ is parameterised as ${B}_{{\rm{c}}2}(T,\varepsilon )={B}_{{\rm{c}}2}(0,\varepsilon ){(1-t(\varepsilon ))}^{s}$ where $t(\varepsilon )=T/{T}_{{\rm{c}}}(\varepsilon )$ is the reduced temperature, ${B}_{{\rm{c}}2}(0,\varepsilon )$ is defined through the relation ${B}_{{\rm{c}}2}(0,\varepsilon )/{B}_{{\rm{c}}2}(0,0)={({T}_{{\rm{c}}}(\varepsilon )/{T}_{{\rm{c}}}(0))}^{w}$, and *s* and *w* are constants. The resulting equation for *g* is

6 $$g({\rm{HTS}})={|\frac{{\rm{d}}{T}_{{\rm{c}}i}}{{\rm{d}}{\varepsilon }_{{\rm{JD}}}}|}_{{\varepsilon }_{{\rm{JD}}}=0 \% }\frac{1}{{T}_{{\rm{c}}}(0)}[\frac{2\,[1+{t}^{2}(0)]}{1-{t}^{2}(0)}+(\frac{st(0)}{1-t(0)}+w)(\frac{qb(0)}{1-b(0)}+n-p-m)],$$

where ${|{\rm{d}}{T}_{{\rm{c}}i}/{\rm{d}}{\varepsilon }_{{\rm{JD}}}|}_{{\varepsilon }_{{\rm{JD}}}=0 \% }$ is the magnitude of the strain dependence of the critical temperature in a single domain at *ε*_JD_ = 0 % and *b*(0) is the reduced field *b*(*ε*) = *B*/*B*_c2_(*T*, *ε*) at *ε*_JD_ = 0%. For the Nb_3_Sn sample *B*_c2_ is parameterised as *B*_c2_(*T*, *ε*) = *B*_c2_(0, *ε*) (1 − *t*^*ν*^*(ε*)) where *ν* is a constant. The resulting equation for *g* is

7 $$g({\rm{LTS}})={|\frac{{\rm{d}}{T}_{{\rm{c}}i}}{{\rm{d}}{\varepsilon }_{{\rm{JD}}}}|}_{{\varepsilon }_{{\rm{JD}}}=0 \% }\frac{1}{{T}_{{\rm{c}}}(0)}[\frac{2\,[1+{t}^{2}(0)]}{1-{t}^{2}(0)}+(\frac{\nu t(0)}{1-{t}^{\nu }(0)}+w)(\frac{qb(0)}{1-b(0)}+n-p-m)].$$

Here we concentrate on identifying and characterising the signature for emergent behaviour, namely the field, temperature and angular dependence of *ε* = *ε*_peak_. We identify the position of the peak by simply fitting the data to a parabola over small strains about the peak. Changes in *ε*_peak_ caused by thermal expansion are at least an order of magnitude smaller than the variations reported here. The thermal expansion of the REBCO and Nb_3_Sn are determined by the CuBe sample holders because of their large cross sectional areas relative to the samples and are <0.018 % and <0.0005 % respectively^46^. Furthermore, the REBCO sample is constrained by the sample holder in two dimensions so the opposite strain dependencies of the critical parameters in the two directions mean that any effect of thermal expansion on *ε*_peak_ is further reduced and can be ignored^33^.

**Figure 6:**
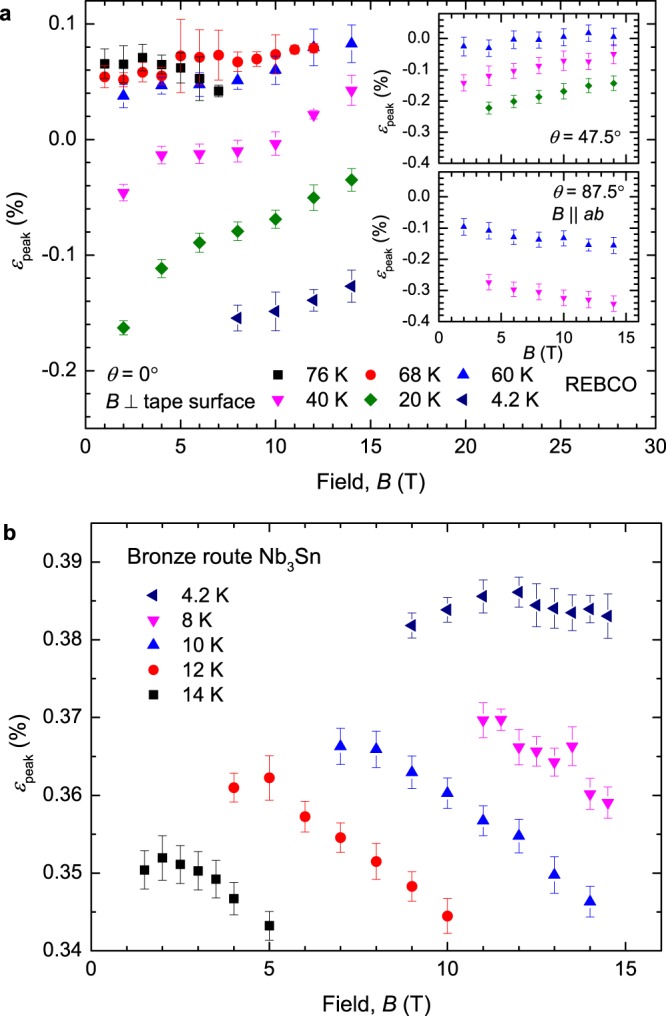
Field and temperature dependence of *ε*_peak_ for (**a**) the REBCO coated conductor and (**b**) the bronze-route Nb_3_Sn. A field and temperature dependent *ε*_peak_ requires competing components and cannot be explained by homogeneous models which predict a constant *ε*_peak_.

**Figure 7:**
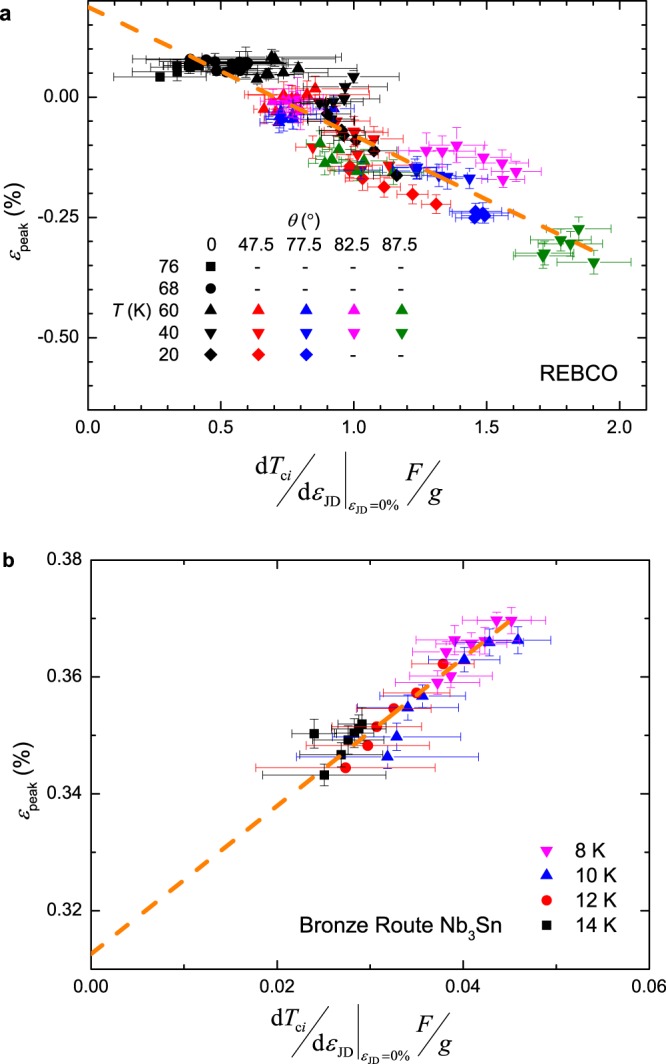
*ε*_peak_ against |d*T*_c*i*_/d*ε*_JD_|*ε*_JD=0%_ *F*/*g* for (**a**) REBCO coated conductor and (**b**) bronze route Nb_3_Sn. The intercept gives ${\varepsilon }_{{J}_{{\rm{cA}}}={J}_{{\rm{cB}}}}$ and the gradient is used to calculate the domain fraction *f*. For both samples the data at 4.2 K are omitted due to heating during measurements in the pool-boiling mode during the resistive transition.

Figure 6 shows the field and temperature dependence of *ε*_peak_ and the insets of Fig. 4 show the double-valued behaviour of ${F}_{{\rm{p}},{\rm{\max }}}{\kappa }_{1}^{2}$ for both samples as expected from bimodal behaviour. In the calculation of *g* the parameters *n*, *p*, *q*, *B*_c2_(0, 0), *T*_c_(0), *s* and *ν* are taken from the experimental results, whereas *m* = 2 and *w* = 2.2 follow the work of Taylor^9^. Figure 7 shows *ε*_peak_ against ${|{\rm{d}}{T}_{{\rm{c}}i}/{\rm{d}}{\varepsilon }_{{\rm{JD}}}|}_{{\varepsilon }_{{\rm{JD}}}=0 \% }F/g$, where the intercept is ${\varepsilon }_{{J}_{{\rm{cA}}}={J}_{{\rm{cB}}}}$ and the gradient is used to calculate *f*. The size of the error bars is predominantly associated with uncertainty in *N*_0_. The data taken in pool-boiling mode at 4.2 K were omitted from this analysis for both samples, due to large uncertainties in *N*_0_ values caused by heating during the resistive transition. A value of ${|{\rm{d}}{T}_{{\rm{c}}i}/{\rm{d}}{\varepsilon }_{{\rm{JD}}}|}_{{\varepsilon }_{{\rm{JD}}}=0 \% }$ is required to calculate *f*. For REBCO we find ${\varepsilon }_{{J}_{{\rm{cA}}}={J}_{{\rm{cB}}}}=0.19\, \%$ and *f* = 0.32 for ${|{\rm{d}}{T}_{{\rm{c}}i}/{\rm{d}}{\varepsilon }_{{\rm{J}}{\rm{D}}}|}_{{\varepsilon }_{{\rm{J}}{\rm{D}}}=0{\rm{ \% }}}=1.6\,{K{\rm{ \% }}}^{-1}$^31^. The value of *f* is within the range of those determined from XRD measurements^47^ given that values of *f* and ${|{\rm{d}}{T}_{{\rm{c}}i}/{\rm{d}}{\varepsilon }_{{\rm{JD}}}|}_{{\varepsilon }_{{\rm{JD}}}=0 \% }$ are expected to change from tape to tape due to different manufacturing conditions. For Nb_3_Sn we find ${\varepsilon }_{{J}_{{\rm{cA}}}={J}_{{\rm{cB}}}}=0.31\, \%$ and *f* = 0.71 for ${|{\rm{d}}{T}_{{\rm{c}}i}/{\rm{d}}{\varepsilon }_{{\rm{JD}}}|}_{{\varepsilon }_{{\rm{JD}}}=0 \% }=0.39\,{K \% }^{-1}$^38^. We note that were the canonical theoretical explanation for the strain dependence of Nb_3_Sn to apply (or if *f* ~ 0.5), *ε*_peak_ would be independent of field and temperature and the dashed line in Fig. 7b would be horizontal.

In this paper we have analysed the relatively small inverted parabolic strain range. At large strains, we find convex behaviour in our data that is also in all our numerical calculations. We also find asymmetry in *J*_c_(*ε*), that can be reproduced in our calculations by including a different strain sensitivity of *J*_c_(*ε*) along the *a*- and *b*-directions^48^. Fitting our data over a larger strain range with multiple components and with different strain sensitivities, introduces more free parameters and will be the subject of future more specialised technical papers.

## Discussion

The bimodal chain model was originally shown to be consistent with the properties of REBCO coated conductors in zero field. Here we have developed it to describe the in-field behaviour of REBCO. Strikingly we have discovered that emergent behaviour also occurs in bronze route Nb_3_Sn as shown by the inset of Fig. 4b and in Fig. 6b. Since the strain dependence of the superconducting materials properties, the upper critical field *B*_c2_(*ε*), the Ginzburg-Landau parameter *κ*_1_(*ε*) and the critical temperature *T*_c_(*ε*), are all derived from *J*_c_(*ε*) they must also be considered weakly-emergent.

The model can explain many of the ‘anomalous’ features of HTS materials in the literature. The field and temperature dependencies of *ε*_peak_ found in published datasets can be explained by values of *f* < 0.5^14,49,50^ and *f* > 0.5^51^. The large variations in *ε*_peak_ between different coated conductors measured in the same experimental setup^16^ can also be explained by differences in *f* caused by the high oxygen mobility at low temperatures in REBCO^52,53^ that also is strain-sensitive^48^. Coated conductors have been manufactured using the Inclined Substrate Deposition (ISD) technique that produce a crystallographic orientation of the *ab*-plane that is rotated by 45° so the [110] direction is along the direction of current flow^30^. In these types of tapes, the strain dependence of both twinned domains is similar so, as with Bi_2_Sr_2_Ca_2_Cu_3_O_x_ conductors which also have unimodal strain behaviour of *J*_c_(*ε*)^54^, there is no competition between the domains^32,55^ and it leads to a weak monotonic strain dependence for *J*_c_. There is also additional evidence in the literature for bimodal behaviour in other LTS materials as evidenced by the double-valued behaviour of ${F}_{{\rm{p}},{\rm{\max }}}{\kappa }_{1}^{2}$^56^.

A deeper understanding of *J*_c_(*ε*) will leverage better strain performance in high field magnet systems through innovative processing of conductors and/or magnet coils. Detwinning HTS materials is already underway to improve strain tolerance of conductors^48^. We suggest that aligning tetragonal Nb_3_Sn may similarly also provide increases in *J*_c_. While the Nb_3_Sn grains in the bronze-route wire reported here are nearly randomly oriented^57^, in Restacked Rod Processed (RRP) and Powder In Tube (PIT) Nb_3_Sn, partial texturing in the <100> and <110> directions respectively occur^58^. We suggest that fabricating conductors that are strongly textured, particularly if high angle grain boundaries could be removed as in the HTS conductors, would be of great interest to test the model presented here further and possibly to achieve much higher *J*_c_ at all strains. At present *J*_c_ in Nb_3_Sn in high fields is less than 1 percent of theoretical limits^59^. The increased technological use of hydrostatic pressure at high temperatures to improve *J*_c_ in both LTS^60^ and HTS superconductors^61^ may encourage using additional strain while operating magnets^62^ and/or innovative means of applying anisotropic stress during conductor or coil processing heat-treatments to encourage the growth of aligned HTS, or aligned tetragonal Nb_3_Sn. While heat-treating coils, one could simply use mechanical stress directly. However high temperature processing more suitable for industry may include putting physical inserts with different thermal expansion coefficients to the coils in say the bore of the coils and removing them after the heat-treatment, or even using electromagnetic stress, produced by putting current through the copper of the coil conductor.

Such understanding of *J*_c_(*ε*) also helps identify the intra- and intergranular microscopic origins of the component parts with opposite strain dependencies in HTS and LTS materials. In HTS, extensive single crystal data directly identifies intragranular properties as one source of competing strain dependencies in the twinned tapes. Although stoichiometric A15 materials can be cubic, technological high field superconductors are generally off-stoichiometric and anisotropic. Anisotropic strain dependencies in Nb_3_Sn are demonstrated by (the limited) single crystal data that show along the (001) direction d*T*_*ci*_/d*ε*_(100)_ = 1.63 K%^−1^^38^ and, similarly to HTS, the hydrostatic strain dependence is much smaller, d*T*_c*i*_/d*ε*_(*hydro*)_ = 40 mK%^−1^ ^34,63^. Given that both REBCO and Nb_3_Sn tapes show that minimising deviatoric strain increases *J*_c_^64^, we conclude that competing intragranular components are important in both REBCO and Nb_3_Sn. All polycrystalline A15 (including Nb_3_Sn) superconductors measured to date^65^ (as well as REBCO reported here, and the superconducting ductile alloy NbTi^66^) have *J*_c_(*ε*) that reaches its peak value when the intrinsic strain is close to zero. As the number of different A15 superconducting materials showing this peak continues to increase, it becomes increasingly untenable that this is because of a fortuitous coincidence between the Fermi energy and a peak in the density of states^35^. Nevertheless for decades, researchers have assumed that measurements on such polycrystalline materials have provided the angularly averaged properties of these materials^34^ without adequate single crystal data. Although the primary origin of the emergent behaviour in polycrystalline Nb_3_Sn is probably associated with the grains and grain boundaries (discussed below)^59^, the canonical explanation for the fundamental inverted parabolic strain dependence of *T*_c_ itself can be challenged since the calculations have only been completed for stoichiometric A15 compounds rather than for the computationally more demanding off-stoichometric, alloyed materials^67^ found in technological wires, and although there is good long-standing evidence for A15 superconductors being strongly coupled BCS superconductors^68^, the Uemura plot presents the possibility that A15 materials may be non-BCS superconductors^69^. For non-BCS superconductors, such as the HTS materials, one simply cannot properly address *T*_c_(*ε*) because there is no reliable explanation for the fundamental mechanism causing the superconductivity.

In polycrystalline Nb_3_Sn, at *J*_c_, dissipation occurs because of flux flow along the grain boundaries where the local superconducting properties are degraded^14^. Current circulating within the grains enables percolative current flow where all grain boundaries of all orientations with respect to the macroscopic current flow contribute to *J*_c_^70^. Hence the effect of Poisson’s ratio will give rise to intergranular contributions to *J*_c_ in polycrystalline materials with opposite strain dependencies because under either compressive or tensile strain, the width of some grain boundaries will increase whilst others will decrease, which will change the coupling between neighbouring grains. Since intergranular superconducting properties are determined by both the grain boundary itself and the grains on either side of the boundary, in general, strain dependencies of both intra- and intergranular components will be important in Nb_3_Sn. Such general considerations of the channels along which flux flows at *J*_c_(*ε*) (e.g. grain boundaries), provide a explanation for why optimum properties are so commonly observed in polycrystalline A15 superconductors close to the unstrained state. Furthermore, to understand the measured properties correctly and to characterise them accurately, these properties must be considered emergent - they are not the angular average of the underlying material, nor are they the properties associated for example with one particular (e.g. the most) degraded region of the material. In HTS materials, it is not clear yet whether low angle grain boundaries or twin boundaries are the location where the flux first moves at *J*_c_, whether flux moves after depinning within channels over-populated by pins within the grains^14^, or after depinning from single pinning sites within grains^5^. Hence whether intra- and intergranular properties must both be considered in HTS, as is the case for Nb_3_Sn, is still open.

To date, the standard literature has continued to describe *J*_c_ in closed form using Eq. (1) even after adding the strain dependence^7,9,10^. However we have found that the mathematical approach required to extend the range of properties included in the functional form of *J*_c_ has depended on whether the new properties are primary or emergent. To achieve an accurate description of strain dependencies, that includes the field and temperature dependence of *ε*_peak_, a different mathematical approach has been required. For as long as only primary properties (*B* and *T*) were included, *J*_c_ was a scaling law of closed form given by Eq. (1). Adding the strain dependence meant replacing the scaling law expression for *J*_c_ by a transcendental equation (Eq. (3)) and restricting the scaling law to be a description of the field and temperature dependence of the component parts alone. Hence the argument that the monotonic strain behaviour (Eq. (4)) of the underlying components is qualitatively different to the inverted parabolic behaviour of the overall *J*_c_ (Fig. 5) has been supported by the change in the structure of the mathematics describing *J*_c_. This underpins Anderson’s rewording of the clichéd description of emergence: ‘the total is… *different to* the sum of the parts’^71^. There is also a change in the important relevant length scale between the primary and emergent properties. The size of the basic building block that determines the field and temperature dependencies of *J*_c_(*B*, *T*) typically has dimensions of a few times a characteristic superconducting length-scale (e.g. the coherence length, the penetration depth or the flux-line-lattice) depending on the nature of the pinning. The properties of a single grain boundary of an LTS material or a single domain for an HTS material are sufficient to characterise the functional form *J*_c_(*B*, *T*) for the whole material. Whereas the basic building block needed to describe the strain dependencies of *J*_c_(*ε*) for the whole material is determined by the microstructure. We need a few competing domains with opposite strain dependencies to understand REBCO or a few competing grains and grain boundaries to understand Nb_3_Sn. Describing the weakly-emergent strain-dependent properties of high field superconductors does not require a very detailed understanding of the complexity of flux pinning, or very precise exponents for the scaling law, any more than describing emergent behaviour in biological systems needs a very detailed understanding of the complexity of the individual insects or birds. In this work, the conclusions and insights into the effect of strain are not sensitive to the precise values of the exponents used in the scaling law (Eq. (1)). In both the superconducting and biological systems, an additional set of equations (e.g. (Eqs (3 and 4)) or local rules leads to a description of the emergent property or overall behaviour.

Amongst the materials physics community, superconductivity is often considered to be the example par excellence for emergence. At the critical temperature (in zero field), the sea of normal electrons collectively condense into Cooper pairs^72^ and bring with them the property of zero resistance^73,74^. The high magnetic field properties are best described by Ginzburg-Landau equations^8^ which include a macroscopic wavefunction as a ground state and together with Abrikosov’s insights^75^, eventually led to the concepts of flux quantisation and flux pinning. We have taken Eq. (1) that describes the field and temperature dependence of the whole material, as the starting point in this paper. However, from a starting point that begins with the sea of normal electrons, even when Eq. (1) is only applied to the flux pinning in the components of these high-field superconductors, it describes emergent behaviour. This has similarities with the classification of the living things considered before. The sociologist considers the behaviour of the individual birds and ants primary, and the behaviour of the flocks and swarms emergent. However, the chemist considers the behaviour of molecules primary, and that of the individual birds and ants emergent^71^. We suggest our work describes the properties of an interesting technologically useful solid-state material that can provide a useful case-study for weak-emergence. The properties of the components are well-defined and relatively simple mathematically (Eq. (4)), as is the relationship giving the competition between the component parts that leads to the overall behaviour (Eq. (3)).

## Concluding Comments

While the approximations that consider high field superconductors as simple homogeneous materials can provide useful engineering parameterisations of *J*_c_ for magnet design, particularly for LTS materials where the field and temperature dependence of *ε*_peak_ is relatively small, we have shown here that this does not describe the underlying science. We have made the observation that *ε*_peak_ varies with field and temperature in both an HTS and a LTS conductor and conclude that any description of similar high field superconductors that attributes the reduction in *J*_c_ under either compressive or tensile strain to the intrinsic averaged underlying strain dependence of any simple component of these materials will not explain the changes in *ε*_peak_ reported here. The evidence for the emergent behaviour in the HTS tape presented in this work follows from a detailed analysis of *J*_c_(*ε*) data and a comparison with single crystal data. Although the strain-dependence in single crystals of Nb_3_Sn is far less detailed than that reported for HTS materials, the A15 single crystal experimental data available do not provide support for intrinsic parabolic behaviour in Nb_3_Sn, with a peak in *J*_c_ observed near zero-intrinsic strain. Hence in addition to the lack of experimental evidence from single crystals supporting the canonical explanation for *T*_c_(*ε*), we add the experimental data in Fig. 6 and the analysis presented here, to make the prima facie case that emergent behaviour also occurs in Nb_3_Sn wires.

It will be a huge challenge to measure and understand the underlying competing components in high field superconductors. Analysis will need to include percolative current flow, and measurements will be required of the anisotropic strain dependence of the superconducting properties of single crystals. In HTS materials, the artificial pinning centres that have produced the highest *J*_c_ values will further complicate understanding the anisotropy of the materials^76^. In LTS materials, there is an obvious need for detailed experimental studies of the anisotropy of off-stoichiometric and alloyed tetragonal single-crystals. To understand LTS materials will also require detailed local measurements of grain boundaries on the scale of the coherence length which will be very difficult. We probably need to develop new tools and new types of experiments for investigating the grain boundaries of polycrystalline metals and may for example use some aspects of the approach that used electron-beam-induced current to look at the electronic properties of grain boundaries in semiconductors, to achieve this^77^. For as long as the flux pinning law (Eq. (1)) included only magnetic field and temperature dependencies, it could be considered a primary law that described averaged property dependencies and fitting parameters. Adding the requirement for the strain dependency of *J*_c_ meant that the scaling law had to be restricted to describing component parts, and a new transcendental equation for the overall behaviour of *J*_c_ was required. This restructuring reminds us of the concern that labelling the properties of an object as emergent, and hence qualitatively different to those of its components, is a subjective judgement. This concern becomes particularly problematic as we consider biological systems and properties such as life or consciousness where agreeing on the essential properties of the components and the overall system is not straightforward^78^. Here we have found that the inclusion of an emergent property is flagged by both a change in the structure of the mathematics and in the important length scales.

We suggest that describing emergence mathematically is not solely a triumph of aesthetics. Using the best category of law (primary or emergent) for the relevant degree of complexity can improve both utility and understanding. The new high field mathematical framework described provides the technological utility of a more accurate description *J*_c_. It also provides a better understanding of how the strain dependence of *J*_c_(*ε*) arises. We suggest that this understanding of *J*_c_(*ε*) as emergent will aid magnet engineers trying to improve high field superconducting materials under strain. This work may also provide a well-defined and simple case-study that can help the broader scientific community develop the language and taxonomy of emergence.
